# Prevalence of major nematodes and human factors that affect infection in the zebra dove in a closed cage system

**DOI:** 10.14202/vetworld.2022.1208-1214

**Published:** 2022-05-19

**Authors:** Watcharapol Suyapoh, Domechai Kaewnoi, Pornphutthachat Sota, Wichaya Thongtako, Sutas Suttiprapa

**Affiliations:** 1 Department of Veterinary Science, Faculty of Veterinary Science, Prince of Songkla University, Songkhla, Thailand; 2 WHO Collaborating Centre for Research and Control of Opisthorchiasis (Southeast Asian Liver Fluke Disease), Faculty of Medicine, Khon Kaen University, Khon Kaen, Thailand; 3 Biomedical Sciences Program, Graduate School, Khon Kaen University, Khon Kaen, Thailand; 4 Tropical Medicine Graduate Program, Faculty of Medicine, Khon Kaen University, Khon Kaen, Thailand

**Keywords:** *Ascaridia galli*, gastrointestinal nematodes, *Geopelia striata*, human factors, *Syngamus* spp, zebra dove

## Abstract

**Background and Aim:**

Roundworms cause infections in the avian population that lead to illness and poor production. The singing zebra dove is an economically important animal in the Indo-Malay region. The prevalence of these parasitic groups in zebra doves is unknown. This study estimated the prevalence and associated human risk factors of gastrointestinal nematode infections in zebra dove farming.

**Materials and Methods:**

A cross-sectional survey was conducted from January to April 2021. The study was conducted on 184 doves in three zebra dove farms. Fecal samples were collected from pooled zebra dove droppings. Major proportions and infection intensity of gastrointestinal nematodes were morphologically identified and morphometrically investigated. Associated human factors were assessed through the interview surveys among farmers.

**Results:**

Results showed that 36.96% of the zebra doves were infected. The primary nematodes were *Ascaridia galli* (34.78%), *Heterakis gallinarum* (6.52%), *Trichostrongylus tenuis* (2.17%), *Syngamus* spp. (4.35%), and *Amidostomum* spp. (2.17%). The primary human factors that contribute to parasitic infection were poor hygiene, food contamination with parasites, and inappropriate deworming.

**Conclusion:**

There was a high prevalence of gastrointestinal nematodes in the zebra dove in the close cage system. Human factors played key roles as risk factors, and improves farming management will help reduce parasitic infections. However, these nematodes may contribute to poor health status and poor productivity of zebra doves. Further extensive studies on clinical signs and pathological changes should be conducted.

## Introduction

The zebra dove (*Geopelia striata*) is commonly raised for singing contests in the Indo-Malay region, including Indonesia, Malaysia, Singapore, Brunei, and Thailand [1]. The national competitions accounts for 50,000-90,000 USD [2-4]. The average price of an individual zebra dove lies between 1000 and 50,000 USD, depending on the singing quality [3]. In Thailand, the large bird farms (200-400 breeding doves) are located in the southern part, particularly in the Narathiwat, Pattani, and Songkhla provinces [5,6]. Zebra doves are associated with several fungal and protozoic pathogens, such as *Cryptococcus neoformans* and *Trichomonas gallinae* [5,7]. However, information on gastrointestinal parasitic diseases in these avian populations and the related factors remain insufficient.

Roundworms cause common parasitic diseases in captive and wild avian species. The common genera of these nematodes include *Ascaridia galli*, *Heterakis gallinarum*, *Trichostrongylus tenuis*, *Strongyloides avium*, *Capillaria* spp., *Amidostomum* spp., *Syngamus* spp., and *Eulimdana* spp. [8-12]. These infections are associated with pathological changes in various organs (gastrointestinal, musculoskeletal, integumentary, and subcutaneous) and respiratory systems [12-18]. These pathologies are related to illness, welfare, egg production, and host immune function [16,18-20]. Various studies have investigated external factors such as ecological, biological, and social conditions. Other important variables include management strategies that promote parasite survival, reproduction, and transmission [21-25].

In this context, a cross-sectional study was designed to estimate the prevalence of gastrointestinal parasitic nematode infection and the related factors, including human activities and farm management. The data from this study will help to develop a desegregated approach to control the transmission of gastrointestinal parasitic nematodes and improve the sanitation of zebra dove farming.

## Materials and Methods

### Ethical approval

This study does not require ethics approval because it used leftover stool specimens.

### Study period and location

The study was conducted from January to April 2021. The samples were collected from birds dropping under the bottom cages. The samples were processed at Department of Veterinary Science, Faculty of Veterinary Science, Prince of Songkla University.

### Experimental design and sampling

**Figure-1:**
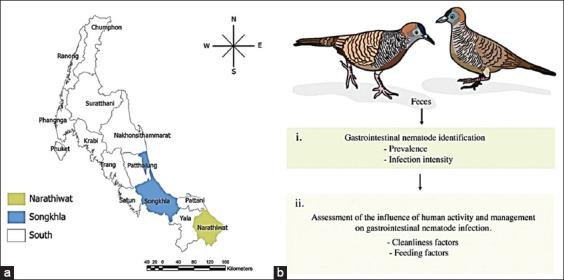
Experimental design. (a) Map of Thailand showing the two provinces where zebra dove feces were collected: Songkla and Narathiwas. [Source: www.diva-gis.org]. (b, i) Study of gastrointestinal nematode prevalence and intensity in 46 flocks of zebra dove. (b, ii) Descriptive assessment of the influences of human activity and management on gastrointestinal nematode detection in zebra doves.

A cross-sectional study was conducted on three zebra dove farms. Pooled fecal samples from 46 flocks (4-6 pet doves per flock) with over 184 birds were collected. A single species, *G. striata*, was assessed from three zebra dove farms in the two provinces Songkla and Narathiwas, Thailand (Figure-1a). All fecal samples were collected in a small container with 10% formalin and directly transferred to the laboratory, where the formalin-ethyl acetate concentration technique (FECT) was applied. Gastrointestinal nematodes were detected by the Department of Veterinary Science, Faculty of Veterinary Science at the Prince of Songkla University (Figure-1b and i). Data on anthropogenic factors affecting parasitic spreading were recorded on a data capture sheet (Figure-1b, ii).

### FECT method and nematode identification

FECT was performed as previously described [26]. Briefly, 10 g of fecal material was suspended in 10 mL of 10% formalin and vigorously shaken. The mixture was filtered through a plastic mold strainer, and the specimens were centrifuged for 3 min at 500× *g*. The supernatant was separated from the sediment and discarded. The fecal sediment was resuspended with formalin and ethyl acetate solution (10 and 4 mL, respectively) and vigorously shaken, followed by centrifugation at 500× *g* for 5 min. The debris and supernatant were decanted, and 2 mL of 10% formalin was added to the remaining sediment, followed by mixing. Then, the volume was measured using counted drops. One drop was used for slide preparation [27]. Nematode eggs were identified morphologically and by size.

### Infection intensity

Infection intensity was calculated from the number of eggs and worms per gram of feces (EPG) [28]. Briefly, one drop of the processed sample was placed on the slide, and eggs and worms were examined and counted under a student microscope (ECLIPSE E200, Nikon, Tokyo, Japan) at 40×. Pictures of eggs and worms were captured using a Nikon advanced upright microscope with a VDO capture digital camera (ECLIPSE Ni-U) (Nikon, Tokyo, Japan). Intensity classification was modified as follows: None=0, 1-99=light (+), 100-299=mild (++), 300-499=moderate (+++), and >500=heavy (++++) [21,28,29].

### Assessment of human factors

The relationships between positive gastrointestinal nematodes in zebra doves and human factors were assessed. Human factors were represented by human activity and management regarding cleanliness, feeding categories, and deworming treatment [30] (Table-1).

**Table 1:** Types of anthropogenic factors and descriptions [30].

Human factor	Description
Cleanliness (poor hygiene)	• Cage cleaning by removal of feces and/or water flushing more than once per week
	• The farmer only removes feces from the cage
	• The farmer removes and cleans feces with water
	• Birds can approach their feces on the cage floor
Feeding (Contamination)	• The farmer provides birds with wild bird rice grain
	• Food remains for more than 3 days
	• Food can be contaminated by other animals such as house rats, cockroaches, house lizards, and ants
	• Water is provided in a bowl and changed frequently
Deworming	• Farmer provides anthelminthic drugs to birds

### Statistical analysis

All results were statistically analyzed using Statistical Package for the Social Sciences (SPSS) version 23.0 (SPSS Inc., USA). Egg size and infection intensity were analyzed and illustrated as mean±standard deviation. Student’s t-test was used to compare pairs, and p<0.05 was considered statistically significant.

## Results

**Figure-2:**
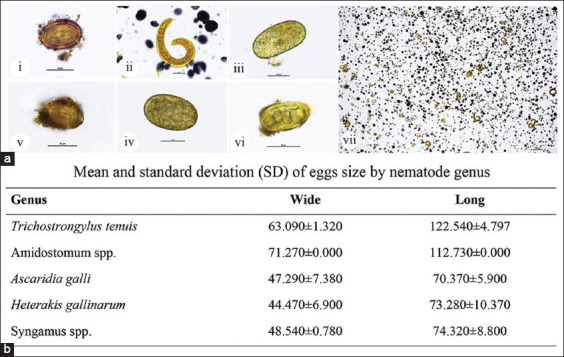
Morphological and morphometric analysis of gastrointestinal nematodes in zebra dove. Representative micrographs of eggs and worms (a), *Ascaridia galli* egg and worm (a, i-ii), *Trichostrongylus tenuis* (a, iii), *Amidostomum* spp. (a, iv), *Heterakis gallinarum* (a, v), and *Syngamus* spp. (a, vi). Heavy infection of *Ascaridia galli* larvae was observed (a, vii). Morphometric analysis of nematode eggs among species (b). (Original magnification, ai-vi=40×, scale bar depicts 50 μm; avii=4×, scale bar depicts 200 μm).

During this study, over 184 birds from 46 flocks of zebra doves were evaluated through fecal specimen examination. Parasite identification was based on morphological and morphometric analysis (Figure-2a and b). The prevalence and intensity of gastrointestinal nematode infection were evaluated in all samples.

### Detection of gastrointestinal nematodes in zebra dove

**Figure-3:**
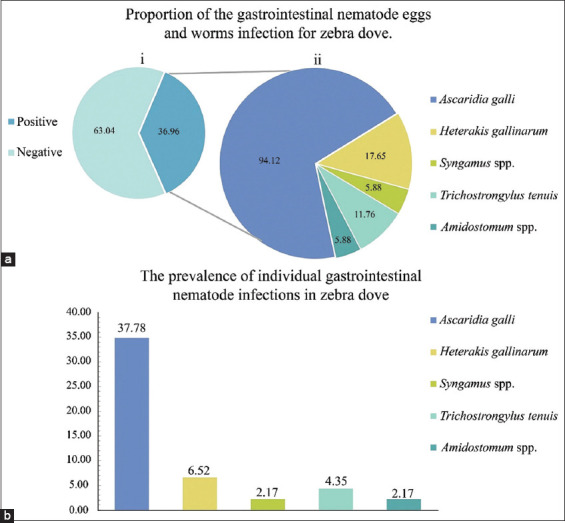
Detection of gastrointestinal nematodes in zebra dove. Proportion of the gastrointestinal nematode eggs and worms (a), pie chart of samples positive and negative for gastrointestinal nematodes (a, i), and proportion of nematode species in positive samples (a, ii). Prevalence of individual gastrointestinal nematodes (b), *Ascaridia galli* (34.78%), *Heterakis gallinarum* (6.52%), *Trichostrongylus tenuis* (2.17%), *Syngamus* spp. (4.35%), and *Amidostomum* spp. (2.17%).

In Asia, the zebra dove plays important economic and socio-cultural roles. To assess its health status by investigating the overall nematode infections, this study initially determined the prevalence of gastrointestinal parasites. Of the 46 flocks, 17 (36.96%) were positive for gastrointestinal nematodes (Figure-3a, i), and 3 (17.65%) specimens showed mixed infections with two or more parasite species (*A. galli*, *H. gallinarum*, *T. tenuis*, *Syngamus* spp., and *Amidostomum* spp.). The parasite distribution was *A. galli* 34.78% (16/46), *H. gallinarum* 6.52% (3/46), and *T. tenuis* 4.35% (2/46), respectively. The lowest frequencies were found for *Syngamus* spp. and *Amidostomum* spp., with 2.17% (1/46) (Figure-3b). Morphological characteristics and morphometric findings regarding nematode eggs and adults are shown in Figures-2a and b.

### Determination of infection intensity

The intensity of infection was assessed using the quantitative examination of nematodes including *A. galli*, *H. gallinarum*, *T. tenuis*, *Syngamus* spp., and *Amidostomum* spp. in pooled zebra dove feces samples (Table-2). For EPG, the highest infection intensity was found in *A. galli* eggs, with a moderate rate (+++; 413.081 ± 1.130.175). In contrast, the infection intensities for *H. gallinarum*, *T. tenuis*, *Syngamus* spp., and *Amidostomum* spp. were low (+), with 19.033 ± 15.241, 6.000 ± 1.838, 12.000 ± 0.000, and 7.300 ± 0.000, respectively. However, there was no statistically significant interaction of infection intensity between parasitic species.

**Table 2:** Infection intensities (means with standard deviation) of nematode eggs (EPG) and worms collected from fecal samples of zebra dove (n=46 flocks).

Eggs/worms	Intensity	Mean±SD
*Ascaridia galli*	+++	413.081±1.130
*Heterakis gallinarum*	+	19.033±15.241
*Syngamus* spp*.*	+	12.000±0.000
*Trichostrongylus tenuis*	+	6.000±1.838
*Amidostomum* spp*.*	+	7.300±0.000

EPG=Eggs per gram, SD=Standard deviation

### Impact of human factors on parasite infection

Among all gastrointestinal roundworms from the three farms, *A. galli*, *H. gallinarum*, and *T. tenuis* were the major gastrointestinal parasites. This raises the question about the risk factors related to gastrointestinal nematode infections in the zebra dove. This study hypothesized that anthropogenic factors such as cleanliness, feeding patterns, and deworming treatment have an impact on the infection in the zebra dove population. To determine the factors associated with the infection, interviews were conducted with the farmers (Table-3). Regarding cleanliness, most farmers (farms No. 2 and 3) cleaned the cages more than once per week. They removed feces with and without water flushing. At the other farm (No. 1), farmers cleaned the cages more frequently (twice per week) using dry sweeping. However, dry sweeping led to unclean cages polluted with feces. Moreover, the farmers are generally not aware regarding the importance of keeping the water and food bowls clean. They frequently left feces in the food and water bowls, which was evident on farms No. 2 and 3. Stored food was cross-contaminated by rats, cockroaches, house lizards, and ants resulting in infections with gastrointestinal nematodes. Most local farmers feed their zebra doves with untreated wild bird rice, which may contain infective stages of parasitic larvae. In most cases, anthelminthic drugs were given for prevention, albeit without the use of food-related dosing instructions. Some farms used Thai local herbs as their primary medical prevention. The treatment frequency in farm No. 2 was monthly, whereas that in farm No. 3 was every 6 months. At farm No. 1, no treatment was performed. Here, it can be implied that inappropriate cage management, poor food hygiene, and the lack of a deworming strategy were related to gastrointestinal nematode infections. However, cleaning the cages is largely limited to avoid stress to the birds, resulting in a low cooing quality.

**Table 3:** Outcomes of the interviews with farmers regarding zebra dove management.

Human factors (human activity and management)		Farm number		

		1	2	3
Cleanliness (poor hygiene)	• Cage cleaning by removal of feces and/or water flushing more than once per week	-	+	+
	• The farmer only removes feces from the cage	+	+	-
	• The farmer removes and cleans feces with water	+	+	-
	• Birds can approach their feces on the cage floor	+	+	+
Feeding (contamination)	• The farmer provides birds with wild bird rice grain	+	+	+
	• Food remains for more than 3 days	+	+	+
	• Food can be contaminated by other animals such as house rats, cockroaches, house lizards, and ants	+	+	-
	• Water is provided in a bowl and changed frequently	-	+	+
Deworming	• Farmer provides anthelminthic drugs to birds	-	+	+

+=Performed, -=Not performed

## Discussion

Zebra doves are economically important in the Indo-Malay region. Parasitic roundworms are common parasites in avian populations and cause symptomatic illnesses, resulting in production and economic losses. This study explored the nematodes present in the feces of 184 zebra doves in closed cage systems. *A. galli*, *H. gallinarum*, *T. tenuis*, *Syngamus* spp., and *Amidostomum* spp. were frequently found.

In the previous studies, the parasites *S. avium* and *Capillaria* spp. were reported as gastrointestinal roundworms present in poultry and birds [9-11]. Moreover, this study detected *A. galli* as a major parasite, which corresponds to the previous studies on poultry and game birds [20,31]. Similar to these results, Sadeghi-Dehkordi *et al*. [32], El-Dakhly *et al*. [33], and Salem *et al*. [34] also reported a high prevalence of *A. galli* and/or *Ascaridia columbae* in pigeons with 90.00%, 87.48%, and 83.30% in the total incidence, respectively.

The highest prevalence of this parasite may be associated with its short life cycle. In addition, its adaptation to their broader environmental conditions allows them to remain viable in the feces and cage for several months [35]. *H. gallinarum*, *T. tenuis*, *Syngamus* spp., and *Amidostomum* spp. were found less frequently, which coincide with the previous studies [36,37]. The co-occurrence of helminths found in this study is usual in most avian species [38,39]. This may be related to the intermediated host and life cycle, which can maintain more than a single nematode species at a time [40]. However, *S. avium* and *Capillaria* spp. were not detected in the feces samples. This was most likely due to the season, the low sensitivity of the diagnostic test, and the low parasitic population [41-43].

This study reflects the average number of individual nematodes present in infected birds. Interestingly, only *A. galli* displayed means infection intensity above the moderate rate (+++). Various factors may contribute to this discrepancy, such as the higher worm burden in natural populations with subsequent infection and reinfection [44]. Thus, continuous surveillance is necessary for raising profitable zebra doves.

Given the high prevalence of gastrointestinal nematodes, this study also explored the anthropogenic factors that may enhance the infection, such as poor sanitation, parasitic contamination in food, and inappropriate deworming. Insufficient cage cleaning can prolong parasitic contamination since the birds constantly pick up infectious stages from the feces. According to Sherwin *et al*. [21], poor hygiene is one of the risk factors for nematode reinfection. Moreover, poor hygiene of food and water, untreated bird rice, food contamination by other animals, and unclean food and water bowls are important factors related to gastrointestinal nematode infection.

Roundworms are common in natural environments and can easily infect animals [45]. Fresh fruits and vegetables can be a source of many parasites, and their consumption represents an important infection route [46]. Furthermore, farms, where other animals are present in the food storage area, showed more helminth infections than those without other animals. This is expected as other animals may play a role as parasite carriers [40,47].

Unclean water bowls can considerably facilitate gastrointestinal nematode infections. In a previous study [22], daily cage cleaning could effectively control gastrointestinal parasites. Interestingly, this study shows an association between dehelminthization and parasitic detection. Most likely, anthelmintic resistance is associated with frequent or inappropriate uses, increasing the resistant nematode population [48-50].

## Conclusion

These findings are essential for management practices and applications, including farmer education, cage and water bowl cleaning, food treatment before use, the prevention of animal carriers, and adequate use of anthelminthic drugs. However, the impacts of gastrointestinal parasitic nematodes on the health status of the birds need to be determined. Further extensive studies should evaluate the clinical signs and pathological changes due to parasitic infection to outline the significance of parasitic diseases and establish effective anthelmintic intervention to overcome this infestation.

## Authors’ Contributions

WS and SS: Conceptualization. WS, DK, WT, and PS: Methodology. WS and DK: Validation. WS and DK: Formal analysis. WS: Investigation. WS: Resources. WS: Writing - original draft preparation. WS and SS: Writing - review and editing. WS: Supervision. All authors read and approved the final manuscript.
